# Identification of ferroptosis, necroptosis, and pyroptosis-associated genes in periodontitis-affected human periodontal tissue using integrated bioinformatic analysis

**DOI:** 10.3389/fphar.2022.1098851

**Published:** 2023-01-06

**Authors:** Shuaifei Pan, Yue Li, Haokun He, Shuguang Cheng, Jiang Li, Janak L. Pathak

**Affiliations:** Guangzhou Key Laboratory of Basic and Applied Research of Oral Regenerative Medicine, Guangdong Engineering Research Center of Oral Restoration and Reconstruction, Affiliated Stomatology Hospital of Guangzhou Medical University, Guangzhou, China

**Keywords:** periodontitis, necroptosis, pyroptosis, ferroptosis, neutrophils, bioinformatic analysis

## Abstract

**Introduction:** Periodontitis is a chronic inflammatory oral disease that destroys soft and hard periodontal support tissues. Multiple cell death modes including apoptosis, necroptosis, pyroptosis, and ferroptosis play a crucial role in the pathogenicity of inflammatory diseases. This study aimed to identify genes associated with ferroptosis, necroptosis, and pyroptosis in different cells present in the periodontium of periodontitis patients.

**Methods:** Gingival tissues’ mRNA sequencing dataset GSE173078 of 12 healthy control and 12 periodontitis patients’ and the microarray dataset GSE10334 of 63 healthy controls and 64 periodontitis patients’ were obtained from Gene Expression Omnibus (GEO) database. A total of 910 differentially expressed genes (DEGs) obtained in GSE173078 were intersected with necroptosis, pyroptosis, and ferroptosis-related genes to obtain the differential genes associated with cell death (DCDEGs), and the expression levels of 21 differential genes associated with cell death were verified with dataset GSE10334.

**Results:** Bioinformatic analysis revealed 21 differential genes associated with cell death attributed to ferroptosis, pyroptosis, and necroptosis in periodontitis patients compared with healthy controls. Gene Ontology (GO) and Kyoto Encyclopedia of Genes and Genomes (KEGG) pathway enrichment analyses revealed that 21 differential genes associated with cell death were related to various cellular and immunological pathways including inflammatory responses, necroptosis, and osteoclast differentiation. Additionally, the single-cell RNA (scRNA) sequencing data GSE171213 of 4 healthy controls and 5 periodontitis patients’ periodontal tissue was analyzed to obtain cell clustering and cell types attributed to differential genes associated with cell death. We found that among 21 DCDEGs, SLC2A3, FPR2, TREM1, and IL1B were mainly upregulated in neutrophils present in the periodontium of periodontitis patients. Gene overlapping analysis revealed that IL-1B is related to necroptosis and pyroptosis, TREM1 and FPR2 are related to pyroptosis, and SLC2A3 is related to ferroptosis. Finally, we utilized the CIBERSORT algorithm to assess the association between DCDEGs and immune infiltration phenotypes, based on the gene expression profile of GSE10334. The results revealed that the upregulated SLC2A3, FPR2, TREM1, and IL1B were positively correlated with neutrophil infiltration in the periodontium.

**Discussion:** The findings provide upregulated SLC2A3, FPR2, TREM1, and IL1B in neutrophils as a future research direction on the mode and mechanism of cell death in periodontitis and their role in disease pathogenicity.

## 1 Introduction

Periodontitis is a chronic infectious disease characterized by plaque biofilm as the initiating factor, gingival inflammation, and the destruction of periodontal supporting tissues. Periodontitis is the leading cause of tooth loss in adults (Montenegro et al., 2020). The global prevalence of periodontitis is estimated to be 20%–50% (Nazir et al., 2020), ranking sixth among the most common diseases. Initially, periodontitis-related inflammation is limited to the gums (gingivitis), but if left untreated, it progresses to periodontitis in a more susceptible population, leading to the destruction of the periodontal membrane and alveolar bone, unstable tooth attachment, and eventual tooth loss (Gopinath et al., 2020). Several studies have found a correlation between periodontitis and cancer, with periodontitis contributing to the development and progression of cancer and inflammatory factors mediating the development of tumors. Several large and medium-sized epidemiological studies and meta-analysis have concluded that periodontal disease may increase the risk of lung cancer (Michaud et al., 2017; Chen et al., 2020), esophageal and gastric adenocarcinoma (Lo et al., 2021). Periodontal treatment led to a significant reduction in the incidence of post-surgical pneumonia in lung and esophageal cancer (Jia et al., 2021). In addition, there is a bidirectional relationship between chronic periodontitis and several systemic diseases. Both periodontitis and liver disease can induce inflammatory responses and lead to the production of inflammatory mediators through which they can interact (Rincic et al., 2022). Sari et al. found that periodontitis and atherosclerosis may have a systemic oxidative stress-enhancing effect (Sari et al., 2022). The coexistence of periodontitis and atherosclerosis increased oxidative stress beyond what was observed alone. With the increasing research on the role of periodontal disease in cardiovascular disease (CVD), periodontal disease may be a risk factor for CVD (Sanz et al., 2020). The reports from the literature found a strong association between periodontitis and type II diabetes, with diabetes promoting the destruction of periodontal tissue and periodontal disease negatively impacting glycemic control (Wu et al., 2020; Santonocito et al., 2022). Therefore, exploring the mechanisms of inflammation regulation during the development of periodontitis can not only prevent and treat periodontitis but also alleviate the development of other diseases to a certain extent.

Current research has found multiple modes of cell death in inflamed tissues. These cell deaths influence the onset and progression of inflammation (Weindel et al., 2022). The methods of cell death include apoptosis, necroptosis, pyroptosis, ferroptosis, *etc.*, each of which has unique molecular characteristics and performs different functions. Necroptosis is a form of programmed cell death that can defend against invasion by certain pathogens. Necroptosis is often involved in the development of inflammatory diseases, and inhibition of necroptosis provides an important target for the treatment of related diseases, especially inflammatory diseases. For example, miR-425-5p improves inflammation and septic liver damage by negatively regulating RIP1-mediated necroptosis (Gu et al., 2020). Excessive viral mimicry produced by endogenous retroviruses triggers Z-DNA binding protein 1-dependent necroptosis, disrupting epithelial barrier homeostasis and promoting intestinal inflammation (Wang et al., 2020). Diabetes and periodontal disease have a mutually reinforcing relationship that can induce severe tissue damage and cell death. Ou et al. (2019) demonstrated that RIP1 and RIP3-dependent necroptosis occurs in the gingival tissue of patients with diabetes-associated periodontitis. Dai et al. (2022) found that RIPK3/MLKL-mediated necroptosis of macrophages contributes to the development of refractory periapical inflammation that can be treated with necroptosis inhibitors. In periodontitis, *Porphyromonas gingivalis* (*P. gingivalis*) destroys oral epithelial cells through RIPK3/MLKL-mediated necroptosis, which further regulates macrophage activation through DAMPs in oral epithelial cells (Geng et al., 2022). Shi et al. (2019) found that necroptosis stimulated the release of DAMP from *P. gingivalis* triggered not only an inflammatory response but also necrotrophic apoptosis in resident periodontal ligament fibroblasts (PDLF). Furthermore, inhibition of cyclin-dependent kinase nine contributes to a reduction in periodontal bone loss and inflammatory response induced by *P. gingivalis* in the periodontal microenvironment through modulation of RIPK3-MLKL-mediated necroptosis (Li et al., 2019). However, the expression pattern of necroptosis-related genes in different cells present in periodontium during periodontitis is still unknown.

Pyroptosis is another mode of programmed cell death discovered in recent years, an inflammatory cell death accompanied by activation of inflammatory vesicles and maturation of the pro-inflammatory cytokines interleukin (IL)-1β/1B and IL-18. Similar to necroptosis, pyroptosis can form NLRP3 inflammatory vesicles, and oligomerized gasdermin D (GSDMD) can also translocate to the cell membrane for perforation (Ding et al., 2016). Inflammatory vesicles can trigger the release of pro-inflammatory cytokines, and their dysregulation can lead to the development of cancer, inflammatory diseases, and neurodegenerative disorders. Toxic factors in diseased periodontal tissues are activated by cysteine, cleaved by GSDMD and IL-1β And IL-18 secretion that aggravate inflammation and tissue damage (Sordi et al., 2021). Bostanci et al. (2009) is team reported that inflammatory vesicles in gingival tissues of patients with periodontal disease were significantly higher than in healthy people. Several studies confirm that pyroptosis plays an important role in periodontitis. GSDMD enhances inflammation and promotes osteoclast formation by increasing IL-1β release, driven by concomitant periodontal ligament stem cell pyroptosis and loss (Chen et al., 2021). Chen et al. (2022a) revealed the key role of pyroptosis in the immune microenvironment of periodontitis by bioinformatics analysis, showing a solid correlation between periodontitis and pyroptosis. Ning et al. (2022) explored the role of pyroptosis in the pathogenesis of periodontitis and the immune microenvironment and identified 14 key pyroptosis-related genes. Xia et al. (2021b) speculated that miR-223-3p in salivary exosomes may regulate GSDMD-mediated pyroptosis by targeting NLRP3 in periodontitis, and therefore detection of miR-223-3p expression in salivary exosomes could be used as an indicator for diagnosis and assessment of the severity of periodontitis. Huang et al. (2020) revealed that the vitamin D analogue eldecalcitol inhibits human gingival fibroblasts (HGFs) pyroptosis *via* activation of the Nrf2/HO-1 pathway. Differentiated embryonic chondrocytes two alleviates periodontal pyroptosis by regulating the expression of NF-κB, cysteine-1, and GSDMD (Oka et al., 2021). *P. gingivalis*-lipopolysaccharide (LPS)/adenosine triphosphate (ATP) induces pyroptosis of HGFs by activating NF-κB/NLRP3/GSDMD signaling. Isoglycyrrhizin attenuates *P. gingivalis*-LPS/ATP-induced pyroptosis by inhibiting these signals (Lv et al., 2021). However, the expression pattern of pyroptosis-related markers in different cells present in periodontitis-affected periodontium should be further investigated.

Ferroptosis is an iron-dependent regulated form of cell death, which is mainly caused by the dysregulation of the balance between intracellular lipid-reactive oxygen species production and degradation when the cellular resistance to oxygen is reduced and lipid-reactive oxygen species accumulate (Li et al., 2021). Numerous studies have shown the involvement of ferroptosis in various diseases such as cancer, inflammation, and neurodegenerative diseases (Tang et al., 2021). Common features between ferroptosis and the pathogenesis of rheumatoid arthritis (RA) indicate ferroptosis modulators as novel targets for RA treatment (Zhao et al., 2022). In addition, periodontitis is a progressive and inflammatory oral disease that leads to damage to the supporting tissues of the teeth. Zhang et al. (2022b) investigated a significant association between ferroptosis-related genes and periodontitis by bioinformatics analysis. Qiao et al. (2022) demonstrated the involvement of ferroptosis in the inflammatory process of HGFs stimulated by *P. gingivalis*-LPS. Wang et al. found that PRDX6 is regulated by NRF2 signaling and alleviates LPS-induced inflammation and ferroptosis in HGFs (Yang et al., 2022). Butyrate disrupts iron homeostasis by activating NCOA4-mediated ferritin phagocytosis, leading to ferroptosis in PDLF (Zhao et al., 2020). Only a few pieces of literature have explored the role of ferroptosis in periodontitis pathophysiology so far. Therefore, the integrated bioinformatics analysis of genes associated with ferroptosis in cells presented in periodontitis-affected human periodontal tissue provides an overall picture of ferroptosis in periodontitis.

Multiple cell death modalities may coexist and interact during periodontitis, and exploring the similarities, differences, and interactions between different cell deaths may provide new research directions to understand the mechanisms of periodontitis pathophysiology. Therefore, the main objective of this study was to identify the expression pattern of the genes associated with necroptosis, pyroptosis, and ferroptosis in different cells present in the periodontium of periodontitis patients through bioinformatic analysis combining single-cell RNA (scRNA) and bulk sequencing data.

## 2 Materials and methods

### 2.1 Bulk sequencing data and microarray data processing

The GSE173078 dataset was taken from the gingival tissues of 12 periodontitis patients and 12 normal individuals. To obtain this data, the SRA file of the GSE173078 dataset was downloaded in Linux using the prefetch command. We used the trim-galore command to remove low-quality bases and adapters for quality control, followed by transcriptome data comparison and featureCount for data quantification using Hisat2, and finally, the count data was obtained (Kim et al., 2019).

The microarray dataset GSE10334 was used as an independent external validation dataset containing gingival tissue data from 63 cases of chronic periodontitis and 64 healthy individuals. The raw data of GSE10334 was downloaded from the GEO database using the GEOquery package (version 2.58.0) (Davis and Meltzer, 2007). According to the GEO database information, the quality control processing on the data was performed before uploading these data. Therefore, we directly used these data for further analysis.

Differentially expressed genes (DEGs) in the GSE10334 dataset were analyzed using a paired *t*-test using the limma R package (Ritchie et al., 2015), and DEGs in the GSE173078 dataset using the DESeq2 R package (Love et al., 2014). DEGs with adjusted *p*-value <.05 and |logFC >1| were considered significant genes. The volcano and heatmap were plotted using the ggplot2 package (version 3.3.5). All datasets participating in this study are listed in detail in Table 1.

**TABLE 1:** The enrolled datasets in the current study.

Datasets	Type	Platform	Sample size (Control/PD)	Cells (Control/PD)
GSE173078	Bulk Sequencing Data	GPL20301 Illumina HiSeq 4000 (*Homo sapiens*)	12/12	—
GSE10334	Microarray	GPL570 Affymetrix Human Genome U133 Plus 2.0 Array	64/63	—
GSE171213	scRNA sequencing	GPL24676 Illumina NovaSeq 6000 (*Homo sapiens*)	4/5	14,552/19,865

### 2.2 Selection of cell death-related genes

Cell death pathways include apoptosis, necroptosis, autophagy, ferroptosis, pyroptosis, and necrosis, which have different morphological and biochemical characteristics. In this paper, we focus on the effects of three cell death modalities, namely ferroptosis, necroptosis, and pyroptosis on periodontitis. Human genes associated with ferroptosis were downloaded from the FerrDb database and 259 genes were found (Supplementary Table S1). The Kyoto Encyclopedia of Genes and Genomes (KEGG) pathway database (https://www.genome.jp/dbget-bin/www_bget?pathway+hsa04217) was used to collect genes associated with necroptosis and found 159 genes (Supplementary Table S2). In addition, 169 genes associated with pyroptosis were also searched in the NCBI database (Supplementary Table S3). Finally, the differential genes obtained from the GSE173078 dataset were intersected with these cell death-related genes to obtain the differential genes associated with cell death (DCDEGs).

### 2.3 GO and KEGG analysis

To investigate the potential biological functions of DCDEGs, Gene Ontology (GO) enrichment analysis and KEGG pathway enrichment analysis were performed by the R package clusterProfiler (Wu et al., 2021). Only terms with FDR <.05 were considered statistically. The top 10 pathways were selected based on the adjusted *p*-value ranking.

### 2.4 Validation of DCDEGs

The obtained DCDEGs were validated in the GSE10334 dataset by searching for DCDEGs in the GSE10334 dataset and the differences in DCDEGs expression between the periodontitis samples and normal samples were calculated and visualized by ggpurb and ggplot2 packages. The genes of p.adjust-values <.05 were considered significant.

### 2.5 Protein-protein interaction (PPI) network

Protein-protein interaction (PPI) network analysis using STRING (https://string-db.org/) to identify interactions between different genes associated with DCDEGs (Szklarczyk et al., 2021).

### 2.6 scRNA sequencing data processing

The scRNA sequencing dataset (GSE171213) from human chronic periodontitis and clinically healthy periodontal tissues was analyzed. This data provides an unbiased assessment of many heterogeneous cells at the single-cell level, thus revealing the complexity of the molecular composition and differences from their counterparts in periodontal tissues. This data was processed in this study as follows, using the R package Seurat v4.0 for quality control, normalization, integration, batch correction, principal component analysis, cell clustering, unified flow approximation, and projection (UMAP) dimensionality reduction (Stuart et al., 2019).

Cells with <200 genes, >2,500 genes, or >5% mitochondrial genes were filtered out during quality control. Gene expression was normalized and further scaled using the “LogNormalize” method. After normalization of the data, 2,000 highly variable genes (HVG) were identified for each sample using the “vst” method. Subsequently, PCA was used to identify significant principal components (PCs) and visualize the *p*-value distribution using the JackStraw and ScoreJackStraw functions. Finally, 15 PCs were selected for UMAP analysis. The FindAllClusters function was used to classify cells into 13 different clusters with a resolution of .2. The FindAllMarkers function with logfc.threshold = .25 was applied to identify DEGs for each cluster. Cell type identification was performed based on the DEGs in each cluster and was checked manually based on a previous study (Chen et al., 2022b).

### 2.7 Immune infiltration analysis

To compare the proportion of immune cells in gingival tissues of patients with periodontitis and healthy individuals, normalized data for genes associated with cell death in the GSE10334 were uploaded to CIBERSORT (https://ciber sort.stanf ord.edu/) (Kawada et al., 2021) and then compared with LM22 (22 immune cell type) gene names of gene signature. CIBERSORT is a deconvolution algorithm, the proportion of 22 immune cell types in each sample and the *p*-value of the deconvolution result for each sample was obtained after calculation. In addition, samples with *p*-value <.01 were considered plausible. Spearman correlation analysis between these genes and the relative proportions of immune cells was calculated and the results were visualized with the heatmap R package (https://CRAN.R-project.org/package=pheatmap).

## 3 Results

### 3.1 Identification of DEGs and DCDEGs

**FIGURE 1:**
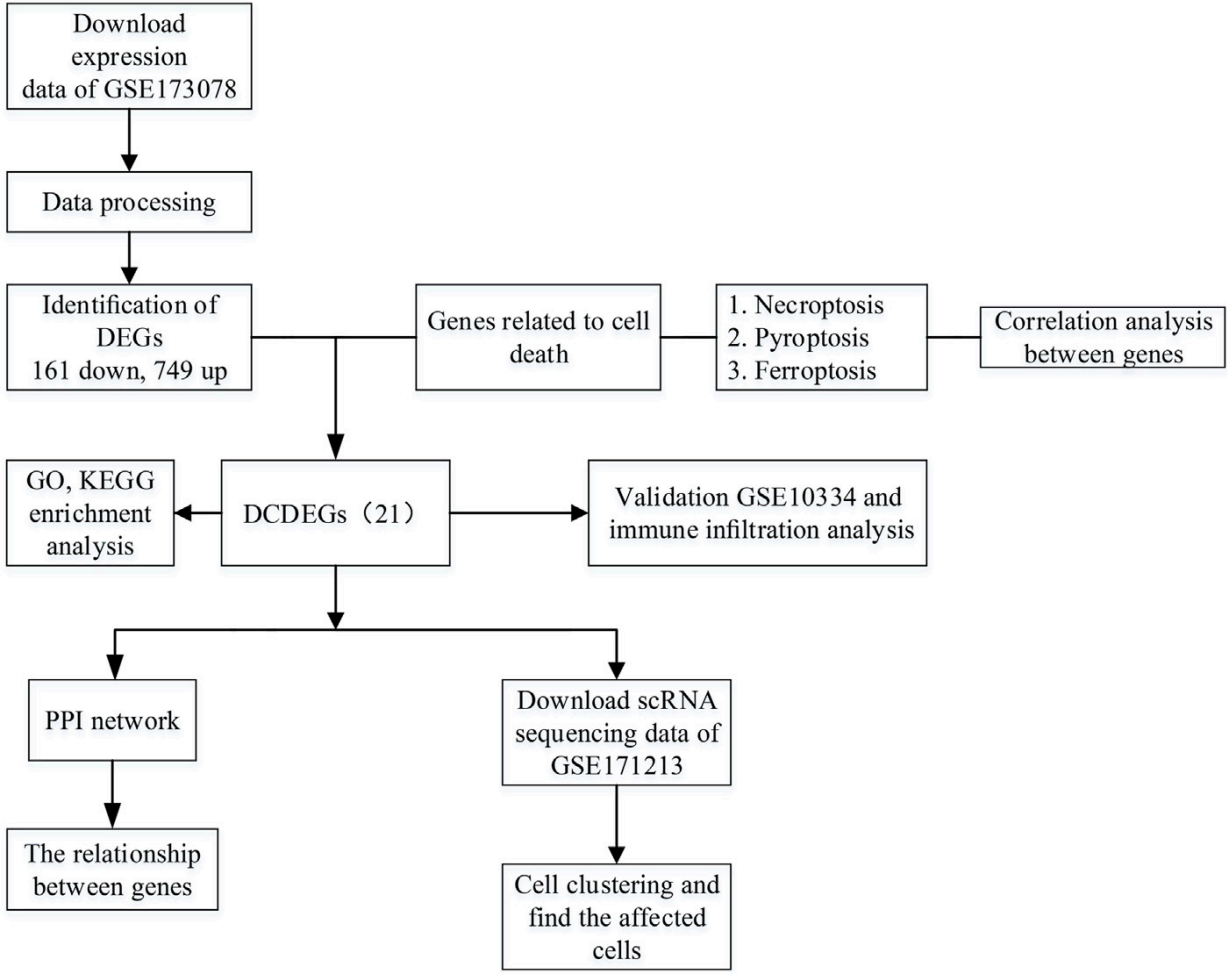
Flowchart of the multistep screening strategy on bioinformatics data, differentially expressed genes (DEGs), differentially expressed genes; differentially expressed cell death-related genes (DCDEGs), differential genes associated with cell death; scRNA, single-cell transcriptomic; PPI, protein-protein interaction.

**FIGURE 2:**
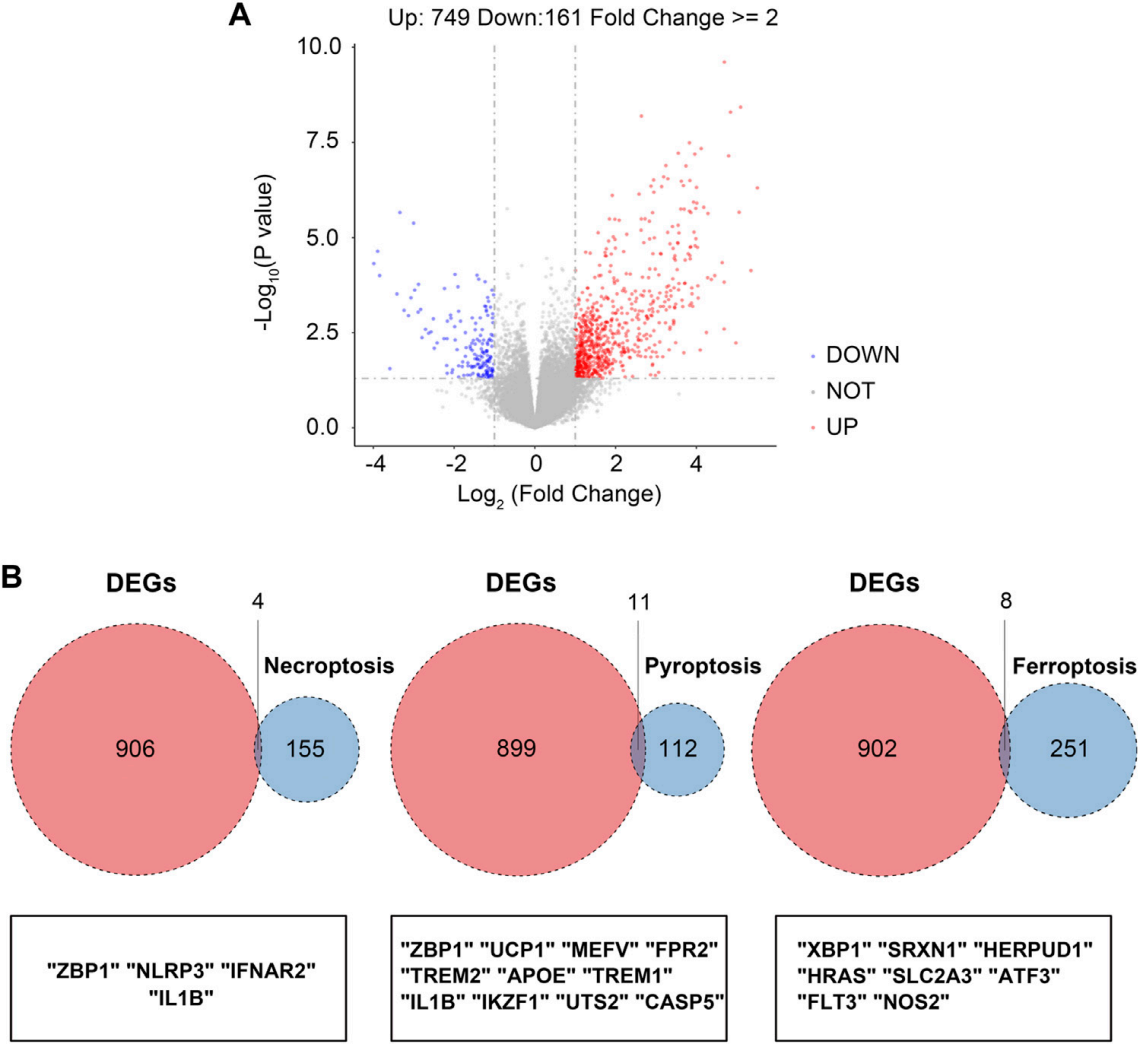
Screening of the differentially expressed cell death-related genes (DCDEGs) in periodontitis. **(A)** The volcano of differentially expressed genes (DEGs) in GSE173078. (periodontitis group vs. control group) **(B)** Venn diagram showing the overlap of genes between DEGs in GSE173078 and cell death-related genes.

**FIGURE 3:**
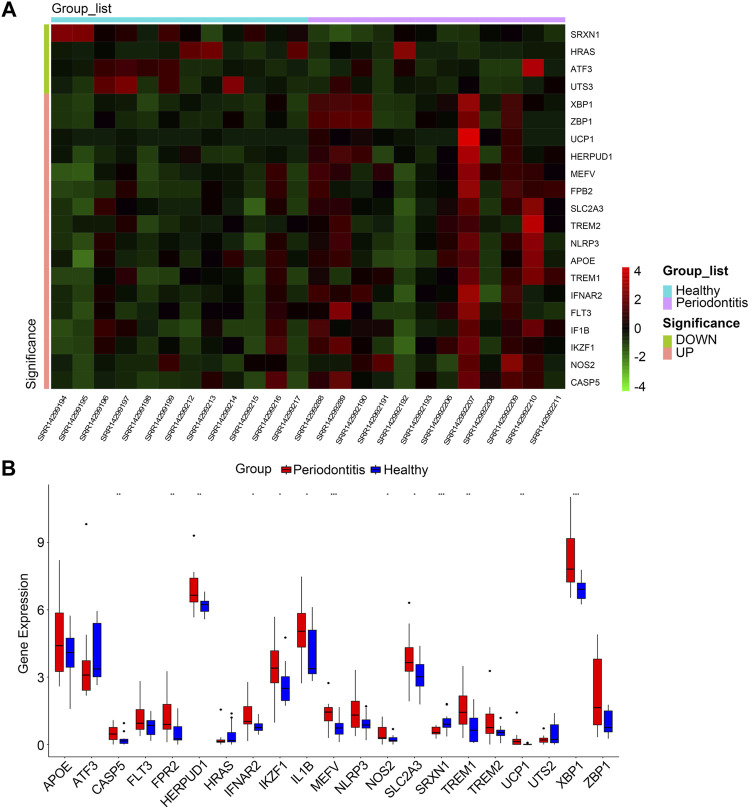
**(A)** Clustered heatmap of DCDEGs. **(B)** The bar graph shows the mRNA expression of 21 DCDEGs in GSE173078. Wilcoxon rank-sum test was used to calculate the *p*-value. Significant difference between the groups, *p < .05, **p < .01, and ***p < .001.

The overall study design is shown in Figure 1. As shown in the volcano plot, a total of 910 DEGs, including 749 up-regulated and 161 down-regulated DEGs, were identified in the dataset GSE173078, based on the comparison of the periodontitis group with the normal group according to the screening conditions of log2FC_cutoff = 1 and pval_cutoff = .05 (Figure 2A). The genes associated with cell death were overlaid with DEGs in GSE173078 and a total of 21 DCDEGs were screened (Supplementary Table S4), including 17 up-regulated and 4 down-regulated. Venn diagram analysis revealed 21 overlapping genes as DCDEGs, including genes such as SLC2A3 and TREM1 (Figure 2B). The heatmap and bar graph of DCDEGs are shown in Figures 3A, B.

**FIGURE 4:**
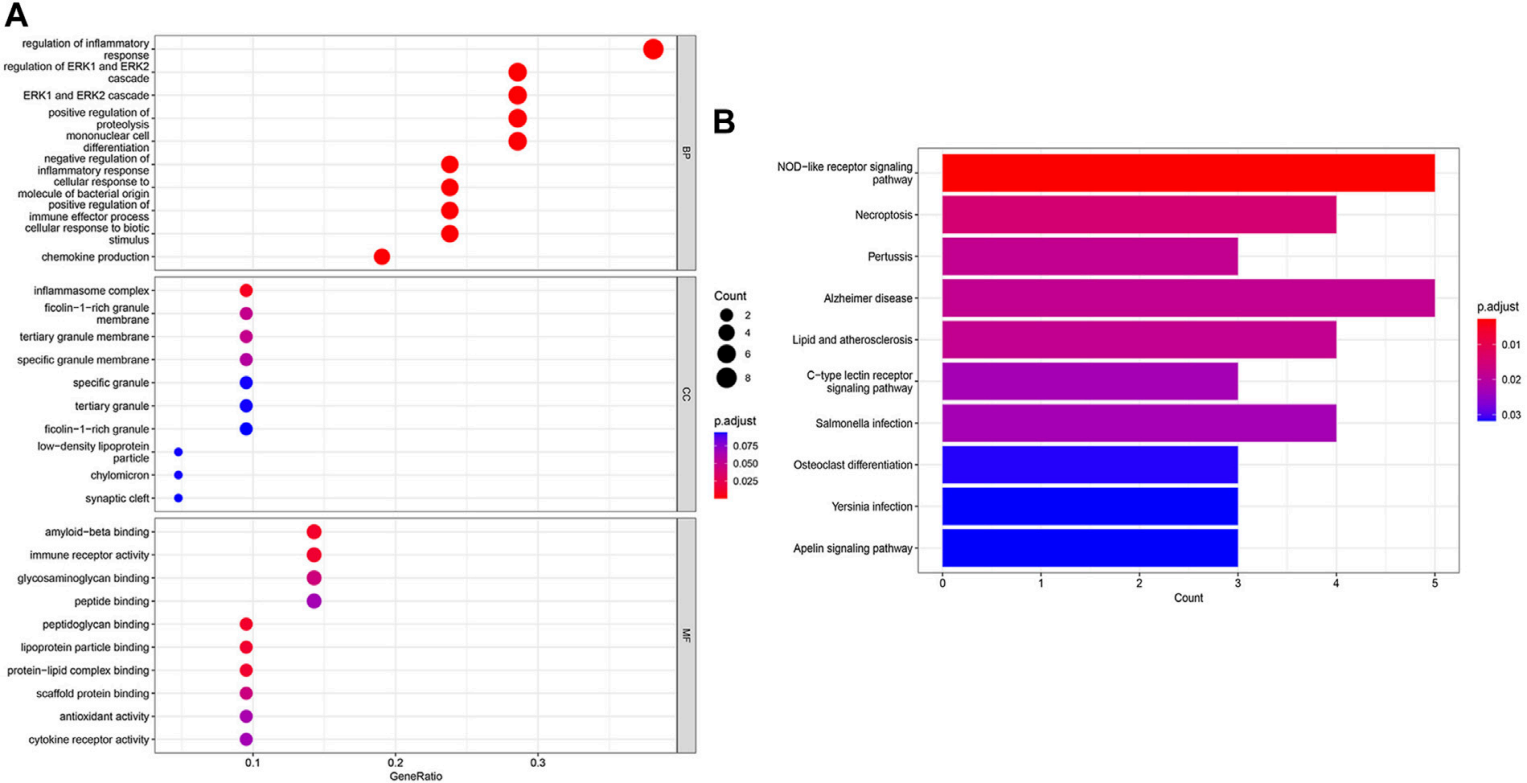
GO and KEGG enrichment analysis of DCDEGs in GSE173078. **(A)** Enriched items in GO analysis. **(B)** Enriched pathways in KEGG analysis. GO, Gene Ontology; BP, biological process; CC, cellular component; MF, molecular function; KEGG, Kyoto Encyclopedia of Genes and Genomes. The top 10 pathways based on p.adjust-value ranking are listed.

GO and KEGG enrichment analyses were performed to identify the functional and related pathways of DCDEGs. A total of 888 significantly related biological processes and 138 KEGG signaling pathways were obtained. GO enrichment analysis revealed enrichment of biological processes in chemokine production, cellular responses to biotic stimulus, positive regulation of cytokine production, inflammatory responses, positive regulation of leukocyte-mediated immunity, regulation of immune effector processes, and regulation of apoptotic signaling pathways (Figure 4A). KEGG analysis showed that DCDEGs tend to be enriched in necroptosis, NOD-like receptor signaling pathway, lipid, atherosclerosis, C-type lectin receptor signaling pathway, *etc* (Figure 4B).

### 3.2 PPI network analysis and relationships between DCDEGs

**FIGURE 5:**
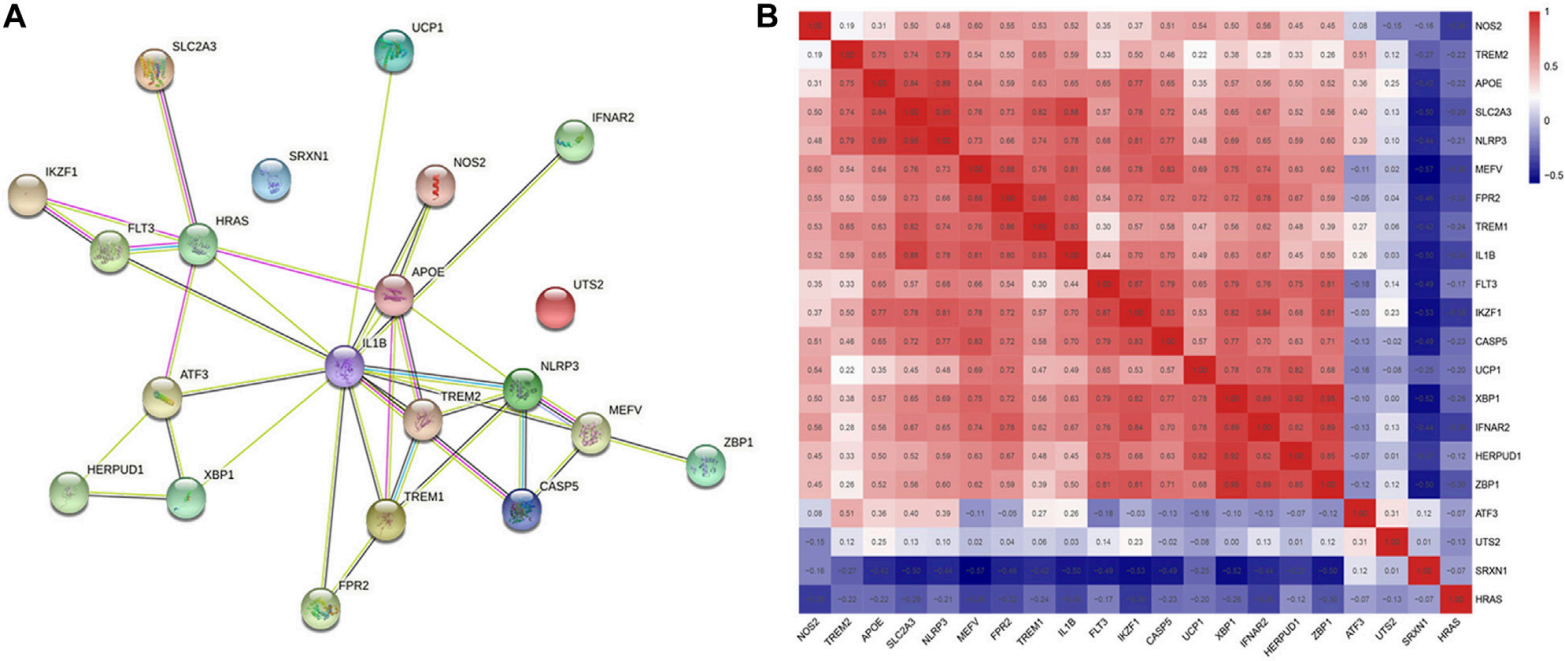
Correlation alanysis of 21 DCDGEs. **(A)** The PPI network of DCDEGs based on STRING. IL1B serves as a hub gene. **(B)** Pearson’s correlation analysis.

The PPI network of 21 DCDEGs was constructed using the STRING database (Figure 5A), which showed the link between these 21 genes. In order to find the correlations between the 21 genes in periodontitis data, we extracted the expression matrix of the 21 genes from the GSE173078 dataset, and then used the cor() function in the corrplot of R package to draw the correlation heatmap of the genes (Zhang et al., 2021). A correlation heatmap was generated and the results showed that the majority of genes were strongly correlated which was used to prepare the exploration of genes related to cells found in Section 3.4 (Figure 5B).

### 3.3 Validation of DCDEGs

**FIGURE 6:**
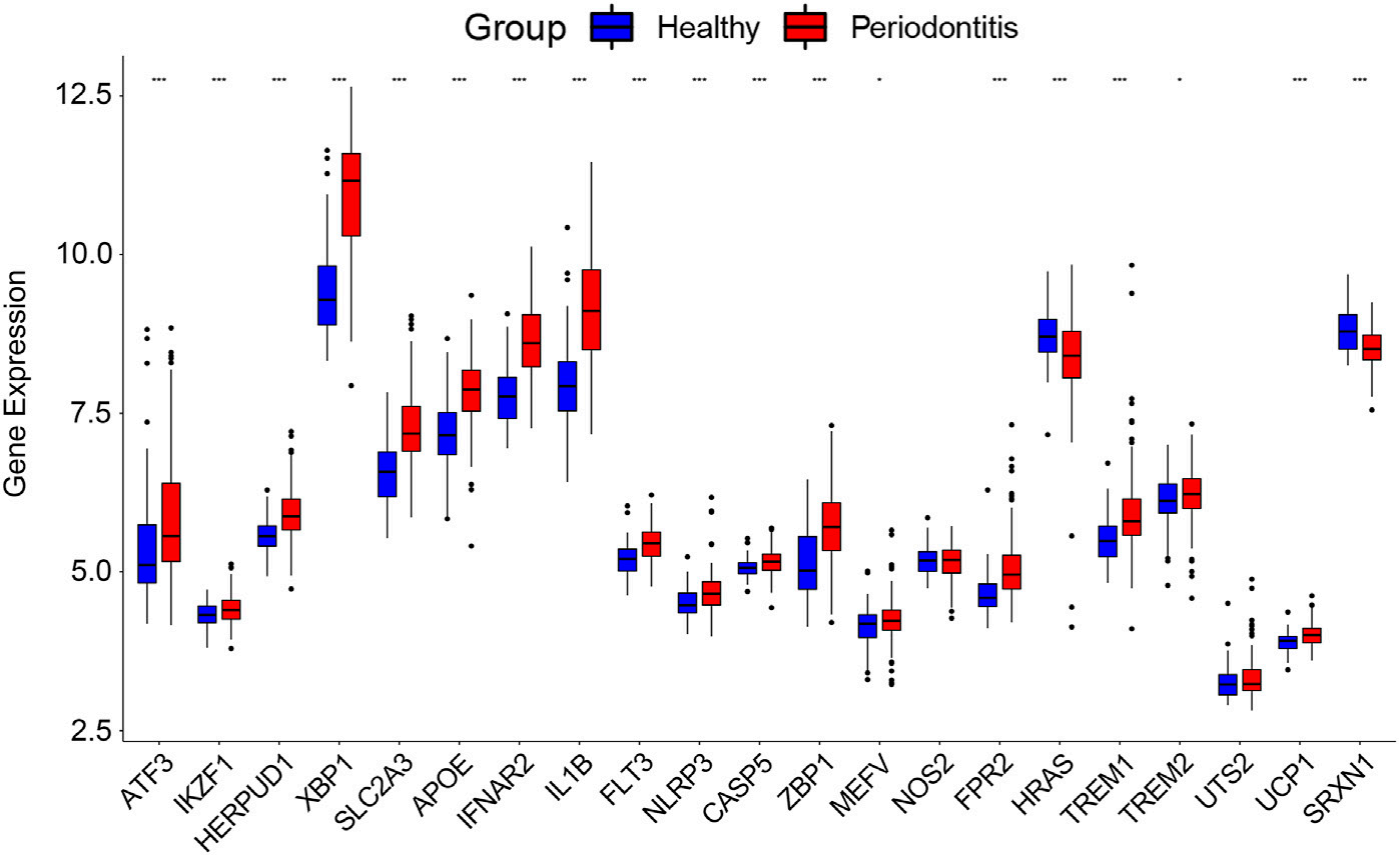
The 21 DCDEGs were validated in the GSE10334 dataset. Wilcoxon rank-sum test was used to calculate the *p*-value. Significant difference, *p < .05, **p < .01, and ***p < .001.

The 21 DCDEGs obtained from the analysis of the GSE173078 data were further validated in the GSE10334 dataset (Figure 6). The results showed that 21 DCDEGs obtained had significant variability in gingival tissue, with SLC2A3 being the most correlated with TREM1, IL1B, and NLRP3 as shown in Figure 5B.

### 3.4 scRNA sequencing data analysis

The scRNA sequencing dataset (GSE171213) from the GEO database was analyzed, with a total of 34,417 cells, including 14,552 cells from healthy control and 19,865 cells from periodontitis patients.

**FIGURE 7:**
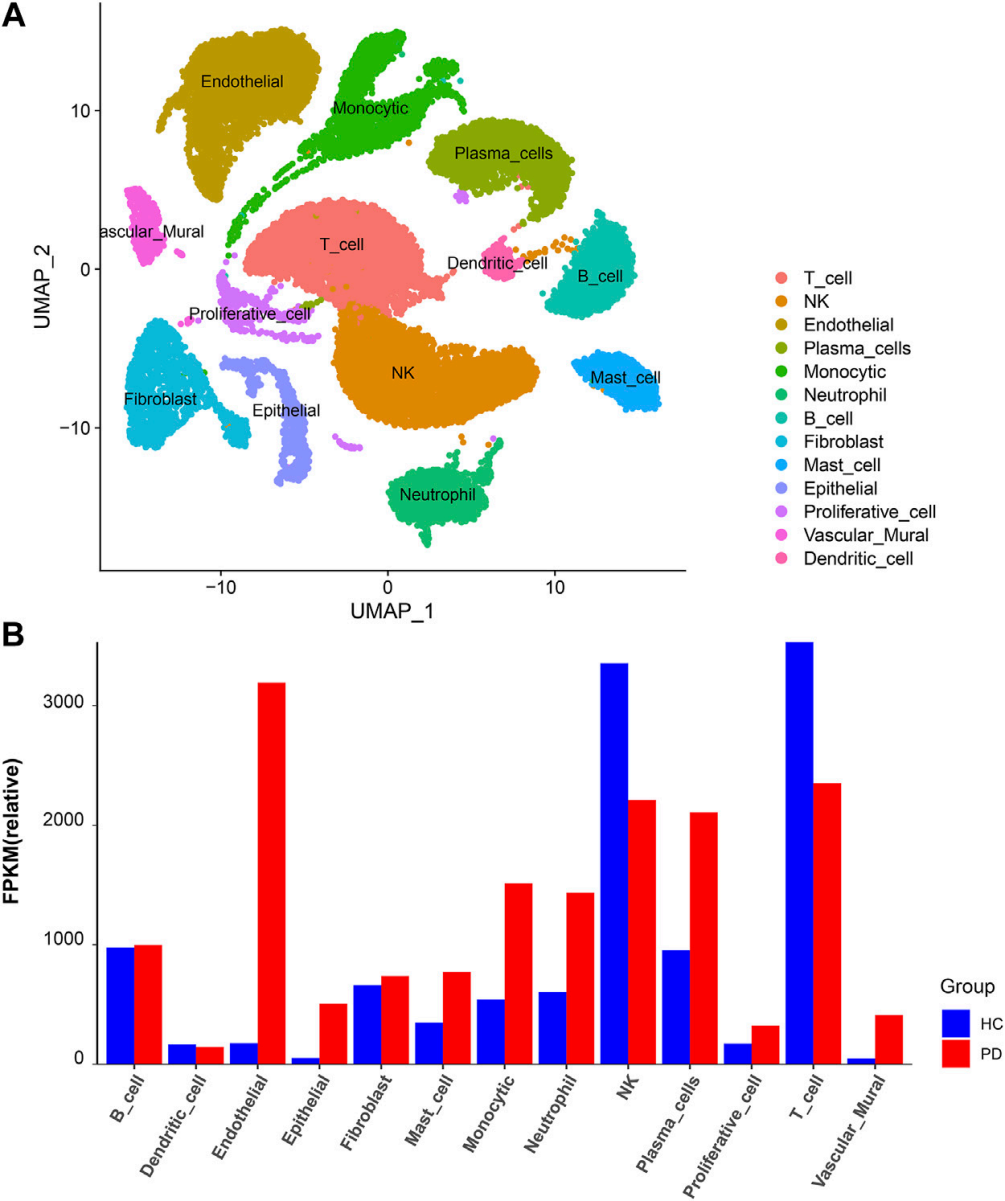
scRNA analysis in periodontitis. **(A)** UMAP projection of 34,417 cells in periodontitis. **(B)** The proportion of each cell cluster in the different sample sets, HC, healthy control; PD, periodontitis.

Based on unified flow approximation and projection (UMAP) analysis, unbiased clustering of cells identified 13 clusters, and marker genes were calculated and then each cluster was annotated according to the marker genes. Specifically, the clusters were divided into 1) T-cell cluster; 2) NK-cell cluster; 3) Endothelial cell cluster; 4) Plasma cell cluster; 5) Monocytic cluster; 6) Neutrophil cluster; 7) B-cell cluster; 8) Fibroblast cluster; 9) Mast cell cluster; 10) Epithelial cell cluster; 11) Proliferative cell cluster; 12) Vascular mural cluster; 13) Dendritic cells (Qian et al., 2021; Chen et al., 2022b) (Figure 7A; Supplementary Figure S1B).

Next, we compared the proportion of each cell cluster in the different sample sets (Figure 7B; Supplementary Figures S1A, S2). From the figures, it can be observed that the proportion of neutrophil clusters, monocytic clusters, and endothelial cell clusters was significantly higher in the periodontitis patient compared with the healthy control. This may be due to the interaction of neutrophils with adaptive immunity that significantly promotes inflammatory events, and the number of these cells was significantly higher in the inflammatory state.

**FIGURE 8:**
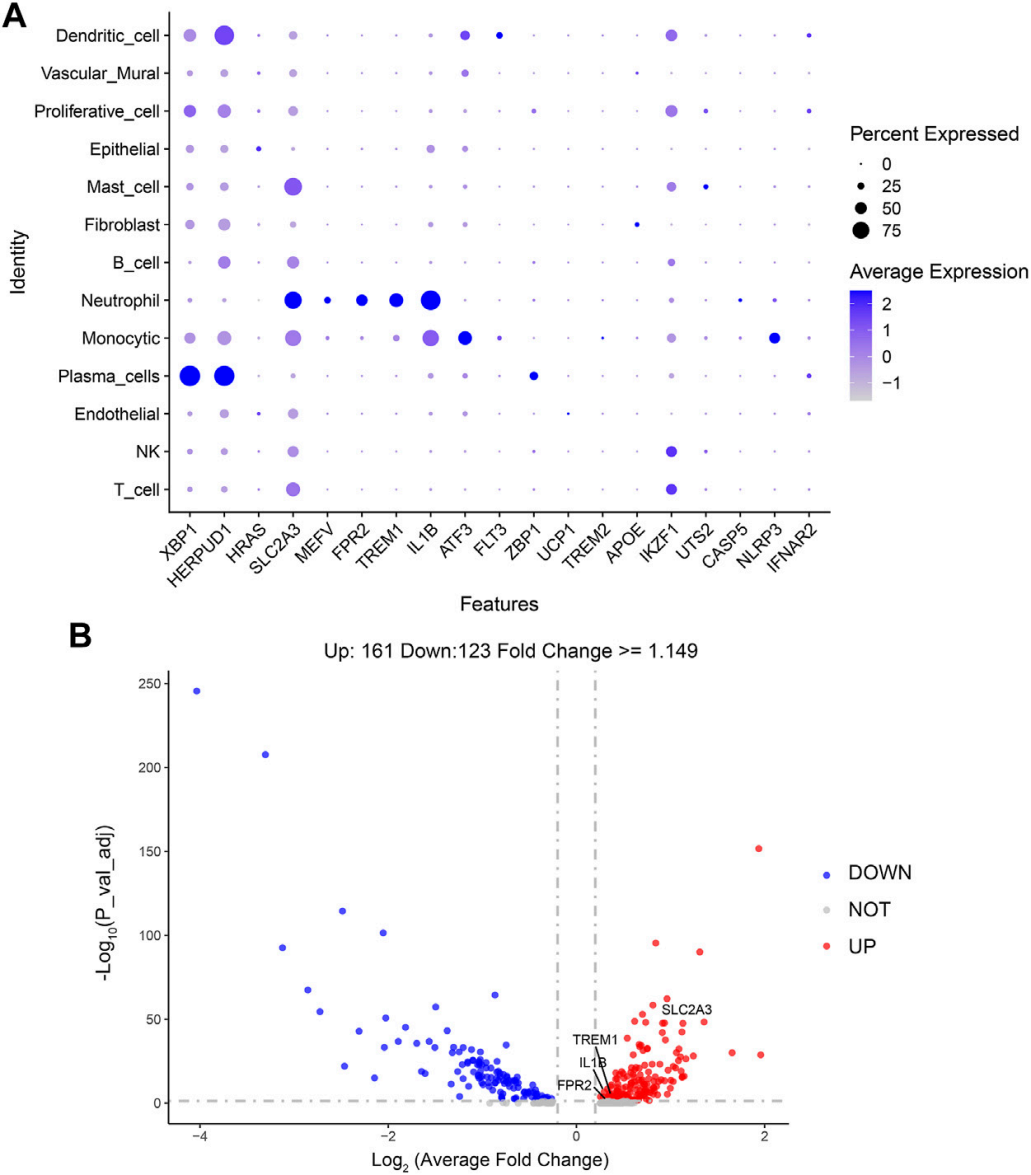
**(A)** Dotplot of DCDEGs. **(B)** The volcano plot of DCDEGs in the neutrophil cluster.

In order to observe which types of cells are affected by the above 21 genes to affect the changes of periodontitis, this paper makes visual maps by using the Dotplot and Featureplot functions in the Seurat package (Figure 8A). From the figure, it can be seen that SLC2A3, MEFV, FPR2, TREM1, and IL1B are significantly distributed in the neutrophil cluster in this dataset, then the difference analysis was done for normal and periodontitis groups in the neutrophil cluster by ggplot2 package (Figure 8B; Supplementary Figure S3), the results showed that SLC2A3, FPR2, TREM1, IL1B were differential, with SLC2A3 being the most significant. In addition, SLC2A3, FPR2, TREM1, and IL1B have a strong correlation as shown in Figure 5B.

### 3.5 Validation of immune infiltration

**FIGURE 9:**
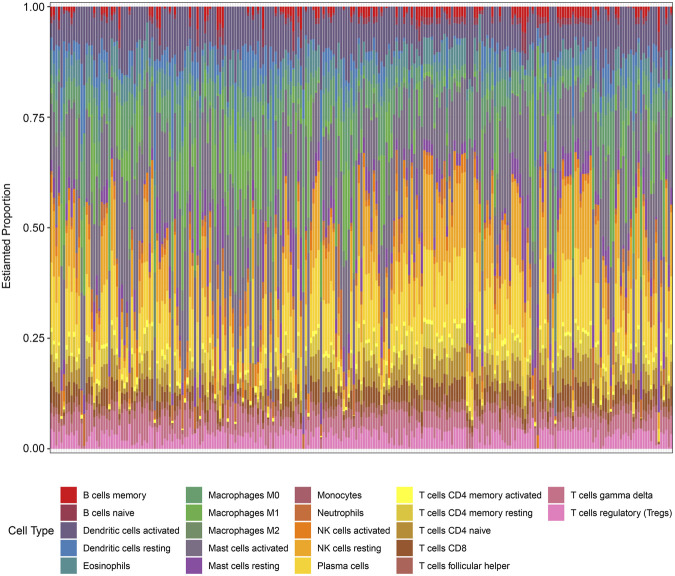
Proportion of 22 types of immune cells infiltrated in gingival tissue of periodontitis patients.

**FIGURE 10:**
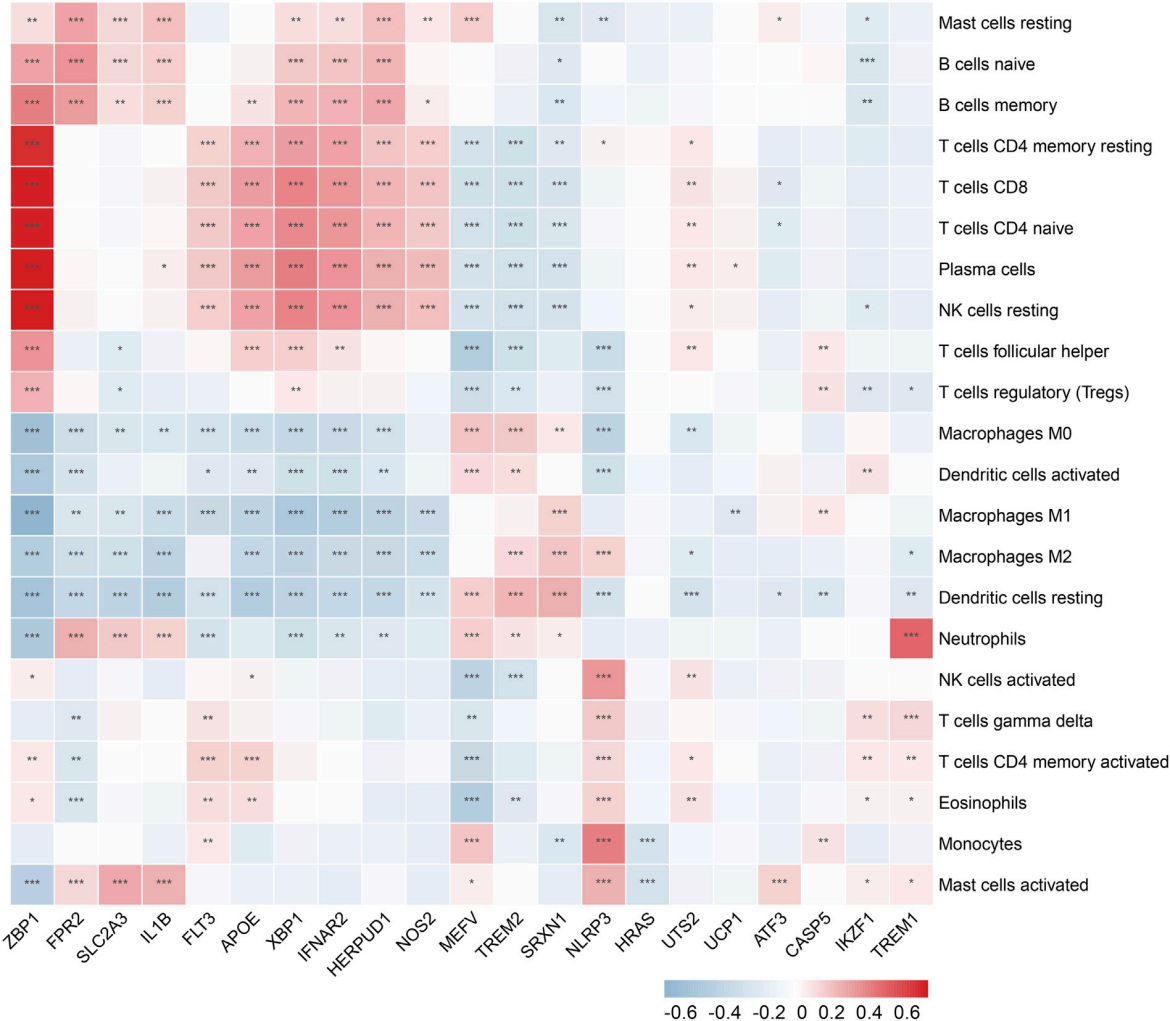
Wilcoxon rank-sum test and spearman correlation were used to explore the correlation between DCDEGs and immune cells by immune infiltration analysis. Significant difference, *p < .05, **p < .01, and ***p < .001.

In the enrichment analysis, we found that DCDEGs were enriched in inflammatory and immune-related pathways. Therefore, in this paper, we used the CIBERSORT algorithm to assess the association between DCDEGs and immune infiltration phenotypes, based on the gene expression profile of GSE10334, to determine the proportion of 22 immune cell types (Figure 9). In order to estimate the correlation between immune cells and DCDEGs to investigate their association and potential interactions, a correlation heatmap of immune cells and DCDEGs was performed (Figure 10). The results revealed that SLC2A3, FPR2, TREM1, and IL1B in DCDEGs were positively correlated with neutrophil infiltration, and thus could confirm the conclusions obtained from the above single-cell data.

## 4 Discussion

Programmed cell death includes apoptosis, necroptosis, pyroptosis, and ferroptosis (Bock and Tait, 2020). The role of apoptosis in periodontitis pathogenicity has been extensively studied and reported in the literature (Song et al., 2017). In contrast, the implication of ferroptosis, necroptosis, and pyroptosis in periodontitis pathogenicity is not been fully understood. Therefore, this study mainly focused on the identification of ferroptosis, necroptosis, and pyroptosis-associated genes in cells present in periodontitis-affected human periodontal tissue using integrated bioinformatic analysis. Bioinformatics analysis of existing mRNA sequencing data, microarray data, and scRNA-seq data from periodontal tissue revealed that SLC2A3, FPR2, TREM1, and IL1B were found to be differentially upregulated mainly in periodontal neutrophils of periodontitis patients compared with the healthy controls. Gene overlapping analysis revealed that IL-1B is a necroptosis and pyroptosis-associated, TREM1 and FPR2 are pyroptosis-associated, and SLC2A3 is ferroptosis-associated. CIBERSORT algorithm analysis confirmed the positive correlation of SLC2A3, FPR2, TREM1, and IL1B with neutrophil infiltration. The PPI network and correlation heatmap analysis revealed the interaction among SLC2A3, FPR2, TREM1, and IL1B. This is the first bioinformatics report describing the upregulation of necroptosis (IL-1B), pyroptosis IL-1B, TREM1, and FPR2, and ferroptosis (SLC2A3) related genes mainly in human periodontium infiltrated neutrophils during periodontitis.

Neutrophils are the most abundant leukocytes in circulation, are closely associated with inflammation and infection in the body, and play an important role in the immune response of the body (Margraf et al., 2022). Neutrophils are involved in several chronic diseases such as atherosclerosis, diabetes, periodontitis, non-alcoholic fatty liver, and autoimmune diseases. In the inflammatory setting, neutrophils are continuously recruited to sites of chronic inflammation and drive the process through the release of serine proteases and the formation of neutrophil extracellular traps, and the activation of other immune cells (Herrero-Cervera et al., 2022). It was found that targeting downstream regulatory element antagonist modulators may be a new therapeutic strategy to reduce excessive neutrophil recruitment in inflammatory diseases (Li et al., 2022a). Neutrophil infiltration is also a common feature of periodontal disease, and it has been demonstrated that stimulation by pathogenic bacteria such as *P. gingivalis* delays neutrophil apoptosis and causes the destruction of periodontal tissue by producing large amounts of tissue-destroying factors in response to pathogenic bacteria. Neutrophils in the oral tissues of individuals with persistent inflammation live longer in patients with chronic periodontitis compared to neutrophils in the oral tissues of healthy individuals (Hajishengallis, 2020). This study found an increase in the number of neutrophils in gingival tissue in periodontitis and an association with genes related to cell death. Therefore, to provide timely relief of inflammation, neutrophil recruitment must be stopped, and migrating neutrophils must be relieved and removed from the affected site (Ansari et al., 2021), which can be achieved by modulating the pathway of programmed cell death (Brostjan and Oehler, 2020).

IL1β, a well-known pro-inflammatory cytokine, is a secretory protein released by a variety of cells including neutrophils. IL1β, an important mediator of the inflammatory response, is associated with a variety of cellular activities, including cell proliferation, differentiation, and programmed cell deaths. In previous literature, intracellular transduction of neutrophil apoptotic signaling is found to mediate tissue inflammation and injury by increasing activation of IL1β through caspase protein expression, which in turn cleaves intracellular matrix proteins that maintain structure and function. IL1β induces pericyte apoptosis through NF-κB activation under high glucose conditions, thereby increasing endothelial permeability in diabetic retinopathy (Yun, 2021). In the pathogenesis of osteoarthritis (OA) disease, an important role was found for the pro-inflammatory cytokine IL1B, and a negative correlation between miR-144-3p and IL1B expression was also observed in OA (Lin et al., 2021). Genes such as FCN1, IL1B, and SERPINA3 are involved in immune cell infiltration and regulate atherosclerosis (AS) through ceRNA (Xia et al., 2021a). IL1β is also importantly associated with neutrophils, and IL1β-mediated antitumor effects depend on infiltrating immunostimulatory neutrophils, and tumor inflammasomes control tumor progression by recruiting neutrophils (Chen et al., 2012). Meher et al. (2018) found that IL1B-induced neutrophil extracellular trap formation (NETosis) promotes experimental abdominal aortic aneurysms. In addition, IL1B plays an important role in periodontitis. Zhang et al. (2022a) searched for key ferroptosis-related genes in periodontitis and constructed an mRNA-miRNA-lncRNA network to deeply explore the pathogenesis of periodontitis, and found the effect of IL1β on periodontitis. Hatipoglu et al. (2022) assessed the effect of B-cell depletion on gingival canal fluid in patients with RA and found that host-regulated therapy in RA reduced local production of IL-1β and MMP-8, thereby alleviating periodontal tissue destruction. Yuan et al. (2022) showed that IL1β, CCL3, and CLEC4E are promising therapeutic targets for peri-implantitis and that these key biological processes and identified genes contribute to the study of peri-implantitis specific to periodontitis. The results of this study and the reports from the literature indicate that the upregulated IL1β in neutrophils infiltrated in periodontal tissue during periodontitis might play a role in pyroptosis and necroptosis of surrounding tissues.

SLC2A3 is a ferroptosis marker involved in transmembrane glucose transport, and current studies on SLC2A3 have focused on cancer. Lin et al. (2022) found that overexpression of SLC2A3 in tumor tissues is associated with poor prognosis in patients with gastric cancer. By immunohistochemical staining, SLC2A3 expression was found to be higher in colorectal cancer tissues than in adjacent non-tumor colorectal mucosal tissues, and SLC2A3 could mediate the immune response by regulating EMT and PD-L1 (Gao et al., 2021). In addition, SLC2A3 has been studied to a lesser extent in other diseases. Dong et al. (2022) studied membranous nephropathy (MN) through the database and screened three genes (TP53, HDAC5, and SLC2A3) associated with MN. In a comprehensive analysis of the role of aging in pulmonary fibrosis based on gene expression profiles, Lu et al. (2021) found that aging-related core genes (SLC2A3, FGA, Hp, and Thbs1) were strongly associated with the development of pulmonary fibrosis. To predict cancer, some scholars have used genes including SLC2A3 to construct models. A study constructed a hypoxia risk model using four hypoxia-related genes, TKTL1, SLC2A3, ALDOB, and ENO3, as a way to assess the tumor immune microenvironment and predict the overall survival of patients with colon adenocarcinoma (COAD), and thus develop hypoxia-targeted drugs for COAD patients (He et al., 2021). In another literature, a prognostic model was developed by using nine genes including SLC2A3, which was effective in improving the prognosis prediction of bladder cancer (Yang et al., 2021). Furthermore, in the immune cell infiltration of oral squamous cells, Han et al. found a significant correlation between SLC2A3 and the infiltration of eosinophils, macrophages, neutrophils, and T helper cells (Han et al., 2021). The role of SLC2A3 in periodontitis pathogenicity and periodontitis-associated cell death has not been reported so far. This study found overexpression of SLC2A3 in neutrophil infiltrated in periodontal tissue during periodontitis. The role of overexpressed SLC2A3 in neutrophil ferroptosis during periodontitis should be further explored.

TREM1 is a member of the triggering receptor expressed in the bone marrow cell (TREM) family and is an immunoglobulin superfamily receptor. Its main function is to recognize foreign antigens and toxic substances, thereby regulating the inflammatory response (Sun et al., 2020). TREM1 is currently extensively studied in cancer and inflammation. Liu et al. (2022) highlighted the importance of anti-inflammatory therapy in thoracic aortic aneurysm and dissection (TAAD) and pointed out that macrophage subsets are a major source of harmful molecules in TAAD and that targeting IL1RN^+^/TREM1^+^ macrophage subsets may represent a promising medical therapeutic approach. Wang et al. (2021) demonstrated that TREM-1 promotes lung injury and inflammation in chronic obstructive pulmonary disease (COPD) mice through the activation of NLRP3 inflammasome-mediated cell scorching, providing a new therapeutic target for COPD treatment. TREM1 is closely associated with neutrophils, and the literature found that TREM1 promotes FOXM1^+^ neutrophil recruitment, has a role in reversing diabetes mellitus, and promotes wound healing *in vivo* (Sawaya et al., 2022). Periodontitis is a chronic inflammatory disease characterized by the destruction of non-mineralized and mineralized connective tissue, and TREM1 knockout mouse models of periodontitis exhibit limited macrophage infiltration, periodontitis lesions, and reduced expression levels of M1 macrophage-related genes in bone marrow-derived macrophages, suggesting TREM1 as a potential gene for the treatment of periodontitis (Wu et al., 2022). This study revealed the upregulation of TREM1 in neutrophil infiltrated in periodontal tissue during periodontitis. The upregulated TREM1 in neutrophils was mainly associated with pyroptosis. Neutrophil pyroptosis is a host immune response to microbial infection. The role of TREM1-mediated neutrophil pyroptosis on periodontitis pathogenicity should be thoroughly investigated.

FPR2 belongs to the family of G protein-coupled receptors and plays an important role in inflammation by interacting with multiple ligands (Corminboeuf and Leroy, 2015). For example, Junaid et al. found that thrombotic inflammatory disease could be alleviated through the AnxA1/FPR2/ALX pathway Ansari et al. (2021). Inhibition of FPR2 expression *via* the NF-κB signaling pathway attenuates the effects of lipopolysaccharide-induced inflammation on trophoblast cells in pre-eclampsia (Li et al., 2022b). To alleviate chorioamnionitis, Li et al. (2020) found that RvD1 attenuated trophoblast inflammation *in vivo* and *in vitro via* the FPR2/PPARγ/NF-κB pathway. FPR2 in bone marrow influences obesity and associated inflammation by regulating muscle energy expenditure, macrophage chemotaxis, and M1 polarization (Chen et al., 2019). In addition, FPR2 is an important chemotactic receptor for neutrophils and its activation causes pro- and anti-inflammatory effects. Sun et al. (2021) found that FPR2 not only directly chemotacticizes neutrophils, but also regulates chemokine production to control chemotactic neutrophil infection. FPR2-induced monocyte and neutrophil chemotaxis and recruitment mediates the effects of neutrophils by regulating macrophage M1 polarization-mediated inflammation and plays a key role in high-fat diet (HFD)-induced obesity and its associated complications (Chen et al., 2019). In the present study, FPR2 was upregulated in neutrophils infiltrated in periodontal tissue during periodontitis and mainly attributed to pyroptosis.

In conclusion, our study showed that SLC2A3, FPR2, TREM1, and IL1B mainly upregulated in neutrophils infiltrated in periodontium during periodontitis. Upregulation of these genes in neutrophils may regulate the death of neutrophils and other cells present in periodontium that might further contribute to periodontitis pathogenicity. Furthermore, SLC2A3, FPR2, TREM1, and IL1B were positively correlated with neutrophils, which may be due to increased expression of certain proteins during intracellular transduction of apoptotic signals from neutrophils, activating SLC2A3, FPR2, TREM1, and IL1B, cleaving intracellular matrix proteins that maintain structure and function, and thus mediating inflammation in periodontal tissue. Therefore, it can be hypothesized that inhibiting the expression of SLC2A3, FPR2, TREM1, and IL1B restores stable neutrophil numbers at the site of inflammation and alleviates inflammation. The rigorous bioinformatics analysis performed in this study provided hints that SLC2A3, FPR2, TREM1, and IL1B are upregulated in neutrophils infiltrated in periodontium during periodontitis and mainly attributed to necroptosis, pyroptosis, and ferroptosis. Our findings should be further evaluated in clinical samples. The mechanism of upregulation of these genes in neutrophils during periodontitis and their role in cell death and periodontitis pathogenicity should be investigated using *in vitro* and *in vivo* periodontitis models.

## 5 Conclusion

Bioinformatics analysis revealed significant differences in the expression levels of 21 DCDEGs between periodontitis and normal gingival tissue samples, and these 21 DCDGEs were mainly attributed to ferroptosis, pyroptosis, and necroptosis. The scRNA sequencing data analysis revealed that among 21 DCDEGs, SLC2A3, FPR2, TREM1, and IL1B were mainly upregulated in neutrophils present in the periodontium of periodontitis patients. CIBERSORT algorithm analysis revealed that the upregulated SLC2A3, FPR2, TREM1, and IL1B were positively correlated with neutrophil infiltration. The findings of our analysis provide future research direction on the mode and mechanism of cell death in periodontitis.

## Data Availability

Publicly available datasets were analyzed in this study. This data can be found here: GSE10334, GSE173078, GSE171213.

## Supplementary material

SUPPLEMENTARY FIGURE S1 scRNA analysis in periodontitis. **(A)** The proportion of each cell cluster in the different sample sets. **(B)** UMAP projection of 3,4417 cells in periodontitis.

SUPPLEMENTARY FIGURE S2 scRNA analysis in the PD compared to the HC. HC, healthy control; PD, periodontitis.

SUPPLEMENTARY FIGURE S3 The difference analysis for normal and periodontitis groups of four genes SLC2A3, FPR2, TREM1, and IL1B.
