# Supplementary figures and images for: Identification of ferroptosis, necroptosis, and pyroptosis-associated genes in periodontitis-affected human periodontal tissue using integrated bioinformatic analysis
Source: Front Pharmacol. 2023 Jan 6;13:1098851. doi: 10.3389/fphar.2022.1098851 (PMC9852864; doi:10.3389/fphar.2022.1098851)

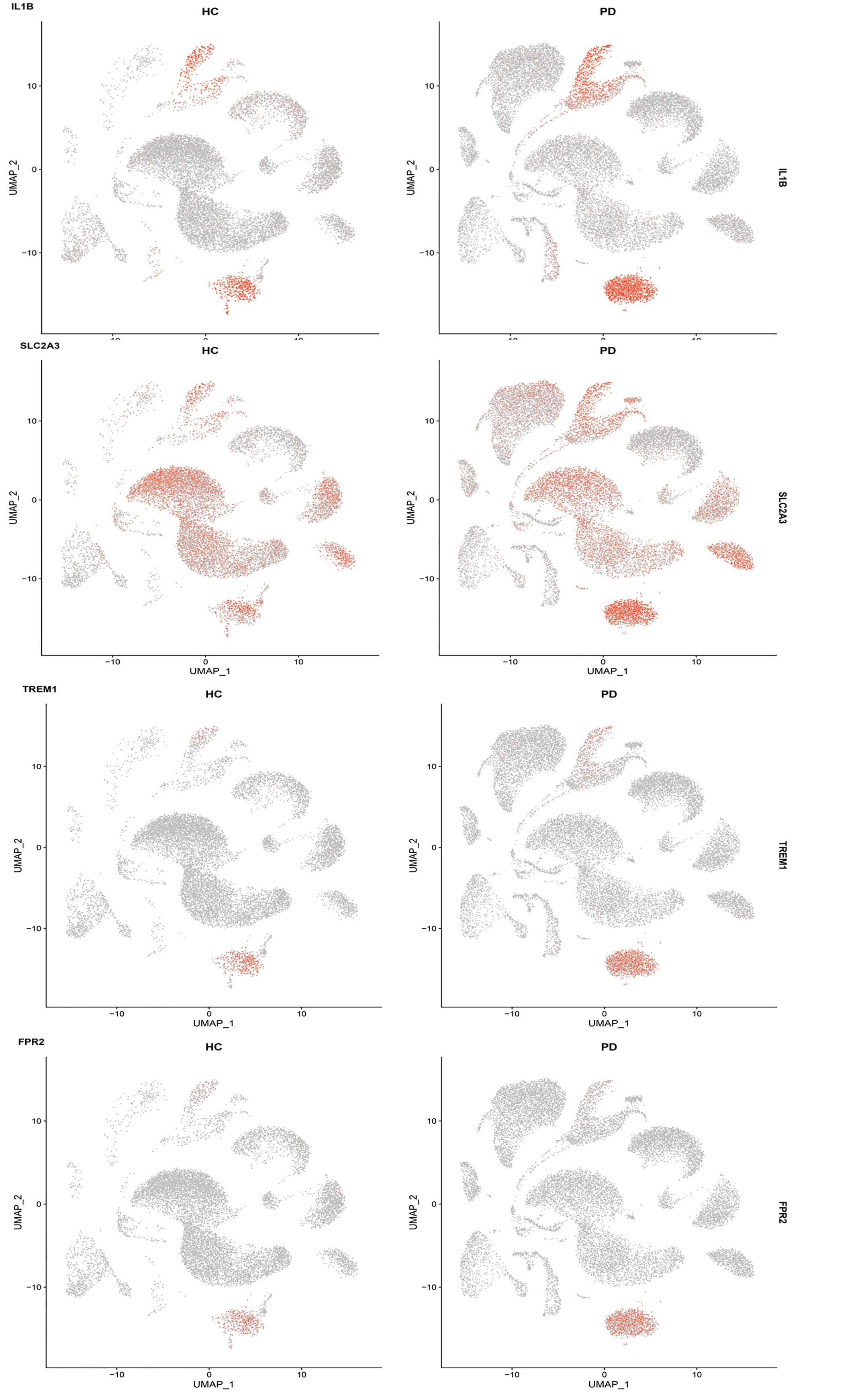

Supplement: Supplementary file 2 [file Image3.JPEG]

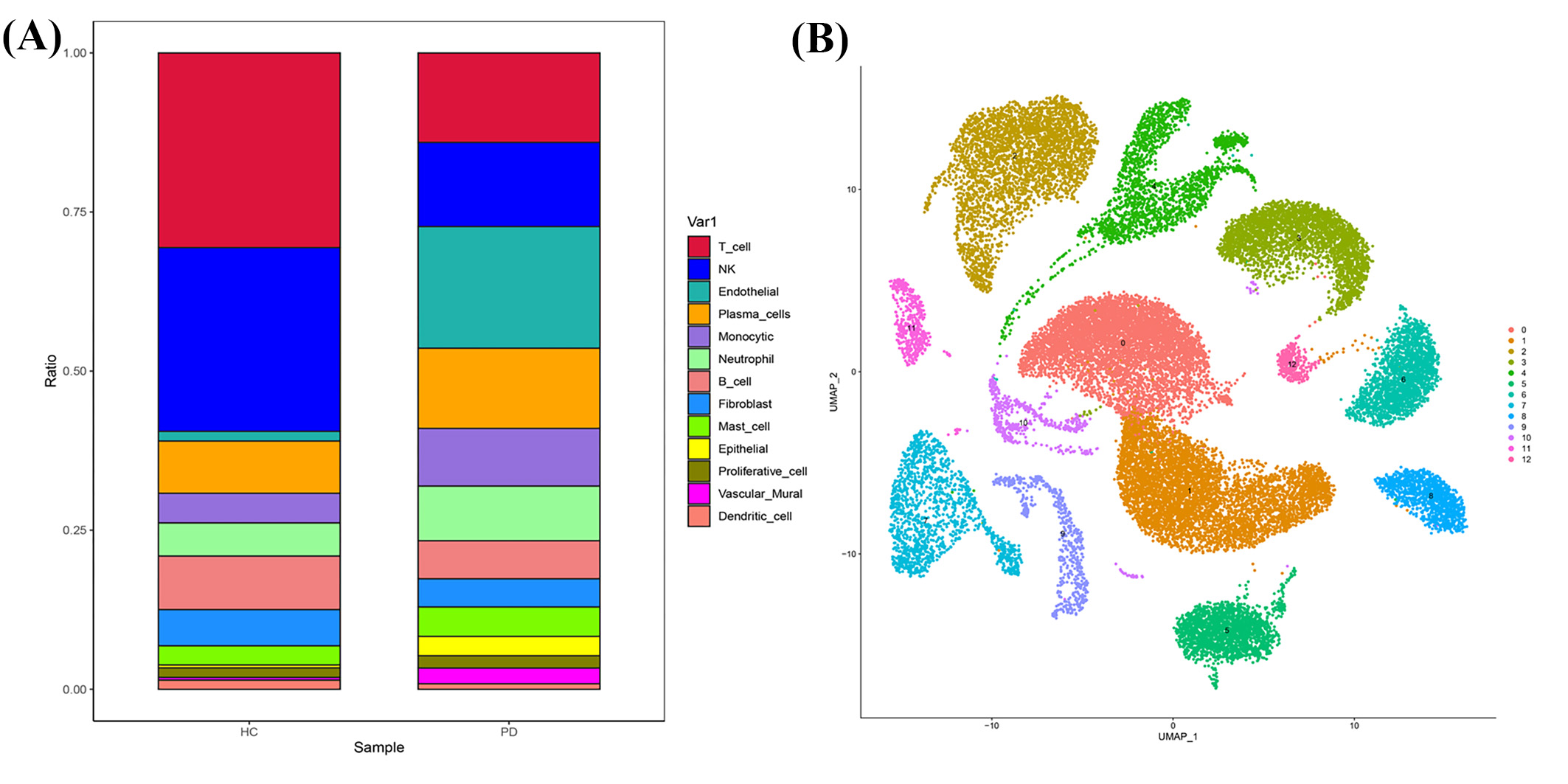

Supplement: Supplementary file 4 [file Image1.JPEG]

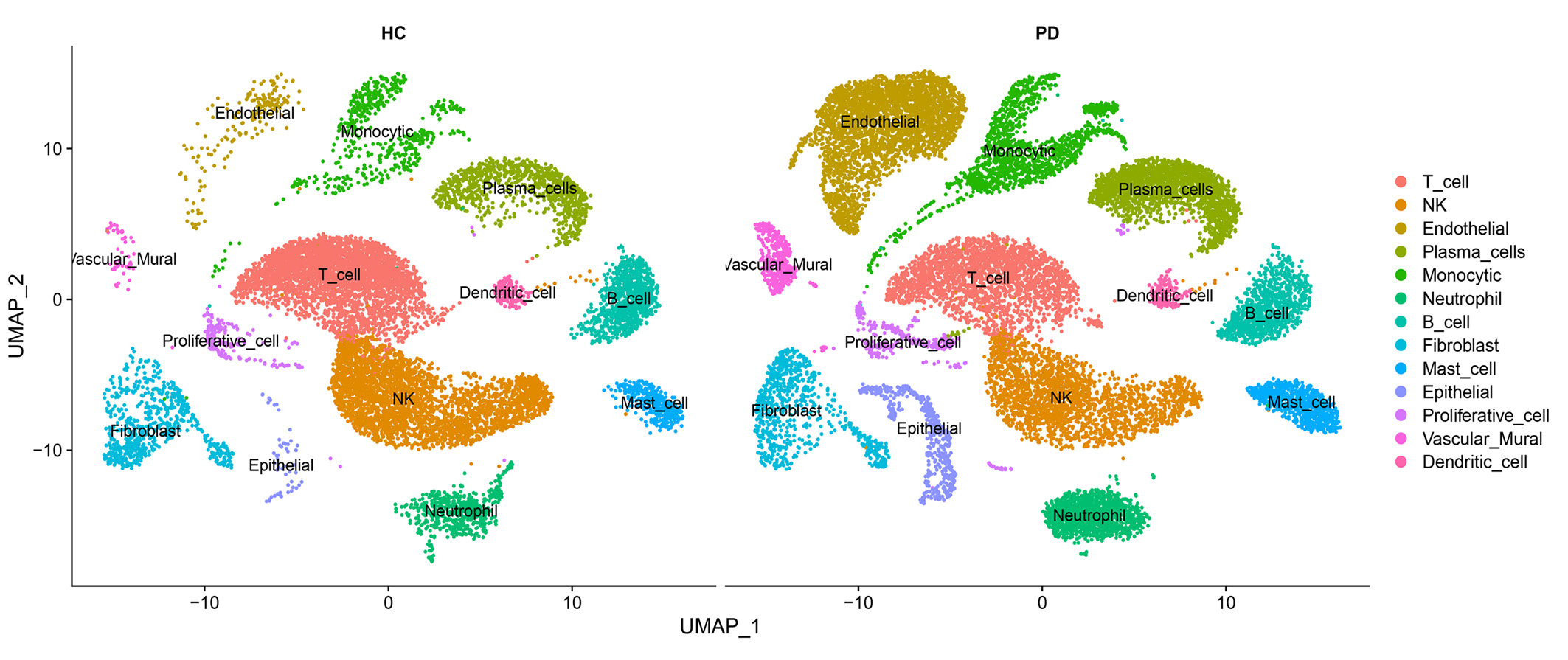

Supplement: Supplementary file 5 [file Image2.JPEG]
